# Microstructure and Piezoelectric Properties of Lead Zirconate Titanate Nanocomposites Reinforced with In-Situ Formed ZrO_2_ Nanoparticles

**DOI:** 10.3390/ma15041389

**Published:** 2022-02-14

**Authors:** Jianhua Li

**Affiliations:** College of Police Equipment and Technology, Chinese People’s Police University, Langfang 065000, China; ljhwjxy@163.com; Tel.:+86-0316-2067120

**Keywords:** PZT, nanocomposites, mechanical properties, piezoelectricity

## Abstract

Lead zirconate titanate (PZT)-based ceramics are used in numerous advanced applications, including sensors, displays, actuators, resonators, chips; however, the poor mechanical characteristics of these materials severely limits their utility in composite materials. To address this issue, we herein fabricate transgranular type PZT ceramic nanocomposites by a novel method. Thermodynamically metastable single perovskite-type Pb_0.99_(Zr_0.52+*x*_Ti_0.48_)_0.98_Nb_0.02_O_3+1.96*x*_ powders are prepared from a citrate precursor before both monoclinic and tetragonal ZrO_2_ nanoparticles ranging from 20 to 80 nm are precipitated in situ at a sintering temperature of 1260 °C. The effects of ZrO_2_ content on the microstructure, dielectric, and piezoelectric properties are investigated and the mechanism, by which ZrO_2_ toughened PZT is analyzed in detail. The ZrO_2_ nanoparticles underwent a tetragonal to monoclinic phase transition upon cooling. The fracture mode changed from intergranular to transgranular with increasing ZrO_2_ content. The incorporation of ZrO_2_ nanoparticles improved the mechanical and piezoelectric properties. The optimized piezoelectric properties (*ε^T^*_33_/*ε*_0_ = 1398, tan *δ* = 0.024 *d*_33_ = 354 pC N^−1^, *k*_p_ = 0.66 *Q*_m_ = 78) are obtained when *x* = 0.02. *T_c_* initially increased and subsequently decreased with increasing ZrO_2_ content. The highest *T_c_* = (387 °C) and lowest *ε^T^*_33_/*ε*_0_ was obtained at *x* = 0.01.

## 1. Introduction

Lead zirconate titanate (PZT)-based ceramics are commonly used in a wide variety of applications, including transformers, transducers, speakers, electric resonators, filters, memory chips, and tactile displays, among others. [1]. However, these materials typically possess poor mechanical properties, i.e., low flexural strength (*σ*_f_) and low fracture toughness (*K*_IC_). This becomes particularly problematic in compositions near the morphotropic phase boundary (MPB), which exhibits excellent piezoelectric properties. High *σ*_f_ and *K*_IC_ are highly sought after in piezoelectric ceramics for high frequency and high-power applications and for situations involving complex mechanical processing [2,3].

In the past few years, several secondary phases have been added to PZT-based ceramics to improve their mechanical properties by adding a secondary phase. These phases can be classified into three types: (1) metal particles such as Ag [4]; (2) fiber-like materials including ZrO_2_ fibers [5,6] and SiC whiskers [7]; (3) oxide particles such as ZrO_2_ [8,9], CuO [3], Bi_4_Ti_3_O_12_, [10] and ZnO [11]. Although these composites significantly improved the mechanical properties of PZT-based ceramics, an unavoidable deterioration in their piezoelectric properties was also observed.

Nanocomposites have recently received significant attention owing to their superior mechanical properties [12]. Reinforcement by second phase particles for PZT was put forward and studied by Niihara and his coworkers [13,14,15,16], and this is an effective way to change from inter-granular fracture to trans-granular fracture in a PZT matrix. The microstructure of a nanocomposite contains a secondary phase, in which nanoparticles are dispersed within the matrix and the grain boundaries. Zirconia nanoparticles are frequently employed as a toughening secondary phase in ceramic matrices. The phase transformation of pure zirconia from the tetragonal (*t*)-phase to the monoclinic (*m*)-phase occurs at approximately 950 °C, accompanied by a volume expansion of 4%, [17] which generates dilatational and shear stresses that impede crack propagation. Thus, the *σ*_f_ and *K*_IC_ of nanocomposites are higher than those of monolithic ceramics [18]. Furthermore, zirconia particles below a critical size stabilize the *t*-phase at room temperature [19]. The existence of nano-sized metastable *t*-phase ZrO_2_ toughens and strengthens ceramics. Nanocomposites with refined microstructures are widely obtained by the in-situ formation of secondary phases.

Moreover, the macroscopic properties of ceramics are directly related to their phase composition, grain size, homogeneity, and microstructure, which are significantly influenced by the method of preparation and the precursor materials. Conventional fabrication techniques typically produce ceramics having coarse and agglomerated particles with a broad particle size distribution resulting in compositional fluctuations and structural inhomogeneities. PZT synthesized via the polymeric precursor method exhibits few compositional fluctuations and very fine monophasic particles with high surface area and crystallinity [20,21].

In this study, by citrate precursor route, Pb_0.99_(Zr_0.54+*x*_Ti_0.46_)_0.98_Nb_0.02_O_3+1.96*x*_ (PZTN) of which Zr/Ti near MPB was chosen to prepare PZT-based transgranular type nanocomposites embedded with both monoclinic and tetragonal ZrO_2_ via in-situ precipitation. The PZT/ZrO_2_ nanocomposites exhibited superior mechanical properties in comparison to monolithic PZT. Moreover, a modest improvement in the piezoelectric properties of PTZ/ZrO_2_ was achieved using an optimal amount of ZrO_2_ nanoparticles. The role of residual stress and other possible reinforcement mechanisms on the mechanical responses of the nanocomposites was also studied.

## 2. Materials and Methods

### 2.1. Sample Preparation

The compositions of the fabricated nanocomposites were Pb_0.99_(Zr_0.54+*x*_Ti_0.46_)_0.98_Nb_0.02_O_3+1.96*x*_ (*x* = 0.00, 0.01, 0.015, 0.02, 0.03, and 0.04). The (Zr_0.54_Ti_0.44_)_0.98_Nb_0.02_O_2.01_ (ZT) precursor was prepared by dissolving C_16_H_36_O_4_Ti, ZrOC_l2_·8H_2_O (99.999%; Sinopharm Chemical Reagent Co. Ltd., Beijing, China) in ethylene glycol. The solution was then mixed with custom-made niobium solutions and heated at 120 °C until viscous transparent sols were obtained. The ZT polymeric precursors thus obtained were baked at 100 °C for 10 days before being calcined in air to obtain white powders, and the polymeric precursors calcined at 740 °C for 4 h to get white ZT powders. Fine PbCO_3_ powder (99.999%) was mixed with the ZT in a molar ratio of 0.99:1 (PbCO_3_:ZT). The mixtures were then ball-milled for 10 h using agate balls and distilled water as the grinding and dispersing medium, respectively. After milling, the slurry was air-dried at 100 °C and calcined at 740 °C for 4 h. The obtained powders were mixed with 7 wt.% poly (vinyl alcohol) (PVA) and die-pressed at 100 MPa. After burning away the binder in a furnace at 740 °C, the green bodies were sintered at 1260 °C for 2 h in closed alumina crucibles containing powders of the same chemical composition to minimize lead volatilization. The sintered bodies were machined into the required dimensions for the analysis of their properties. Electrodes were formed by applying silver paste to both sides of the disc samples (diameter, 20 mm; thickness, 1 mm), which were then fired at 820 °C for 15 min. Poling was performed in silicon oil at 120 °C for 30 min with an electric field of 3.0 kV mm^−1^. All electrical measurements were taken 24 h after polishing.

### 2.2. Characterization

The bulk density of the samples was determined in distilled water using the Archimedes method. The crystalline phases in the sintered samples were examined using X-ray diffraction (XRD, D/MAX-2500X, Japan Science Corporation, Tokyo, Japan) with Cu K_α1_ radiation in the 2*θ* range of 20–70°. The morphology of the nanocomposites was observed by scanning electron microscopy (SEM, XL30E, Phillips, Amsterdam, The Netherlands). Elemental analysis of the nanocomposites was performed using energy-dispersive X-ray spectroscopy (EDS, Linkisis-300). Detailed microstructural characteristics were examined using transmission electron microscopy (TEM, Tecnai G2 F20, Phillips, Amsterdam, The Netherlands).

The dielectric properties were obtained by measuring the capacitance and dielectric loss at 1 kHz at room temperature using an LCR meter (HP 4294A). The Curie temperature (*T*_c_) was determined from a plot of the temperature vs. dielectric constant at 1 kHz. The piezoelectric constant (*d*_33_) was measured using a quasi-static piezoelectric *d*_33_ meter (ZJ-3d, Institute of Acoustics Academic Sinica, Beijing, China). Using the LCR meter, the electromechanical coupling coefficient (*K*_p_) and mechanical quality factor (*Q*_m_) were calculated using the resonance–antiresonance method based on IEEE standards and the following equations [22]:

(1) $$K_{p}={\left[\frac{\eta_{1}^{2}-[1-{\left(\sigma^{E}\right)}^{2}]}{2(1+\sigma^{E})}\times \frac{f_{a}^{2}-f_{s1}^{2}}{f_{a}^{2}}\right]}^{1/2}$$

(2) $$Q_{m}=f_{a}^{2}{\left[2\pi R_{f}Cf_{r}(f_{a}^{2}-f_{r}^{2})\right]}^{-1}$$

(3) $$\sigma^{E}=\frac{5.332f_{r}-1.867f_{s1}}{0.6054f_{s1}-0.1910f_{r}}$$

*η*_1_ = 1.867 + 0.6054*σ^E^* (4)

where *f_a_* is the anti-resonant frequency, *f_r_* is the resonant frequency, *f_s_*_1_ is the first overtone resonant frequency, *R_f_* is the resonant impedance (Ω), *C* is the electrical capacitance (F), *σ^E^* is Poisson’s ratio (superscript “*E*” indicated short-circuited boundary conditions), and *η*_1_ is the frequency constant of a disk resonator. The impedance vs. frequency spectra of the ceramics were obtained at room temperature with a sweeping frequency ranging from 150 Hz to 215 kHz.

## 3. Results

### 3.1. PZT/ZrO_2_ Nanocomposite Ceramics

#### 3.1.1. ZT Precursor with Pb Vacancies

**Figure 1:**
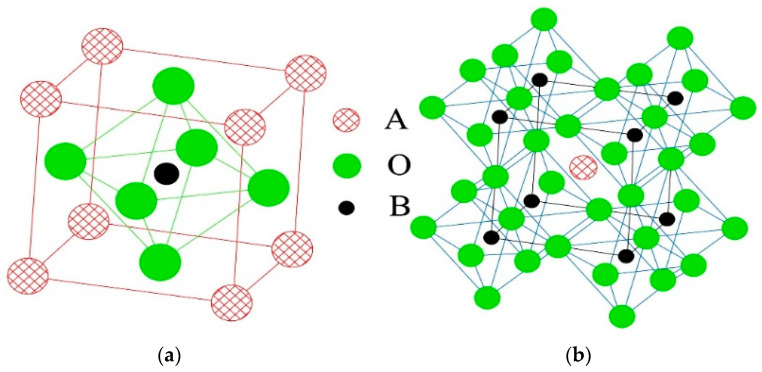
Perovskite-type crystal structure: (**a**) Cubic unit cell; (**b**) Oxygen octahedral structure.

**Figure 2:**
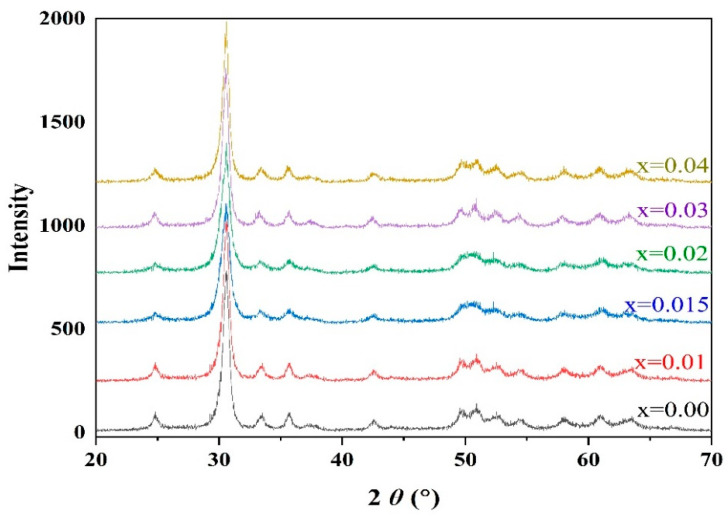
XRD patterns of ZT powders synthesized at 740 °C.

The ZT oxide solid solution of B-site ions within perovskite-type PZT was first synthesized using the precursor method [16,18]. The A-sites were vacant at this stage (Figure 1). The positions and heights of the peaks in the XRD patterns of the ZT precursor powders (Figure 2) were consistent with those of the standard JCPDS XRD data for ZrTiO_4_ (JCPDS card No. 84-1018), confirming the successful synthesis of monophasic precursor powders with the ZrTiO_4_ structure.

The synthesis of perovskite-type PZT powder is typically achieved by a solid-phase reaction; however, intermediate reactions and PbO volatilization tend to occur during this process. Consequently, fluctuations and inhomogeneity in the chemical composition of the PZT powder are often observed owing to the presence of small amounts of intermediate phase PT or PZ, which impact the electrical properties of the PZT ceramic. Additionally, PZT powder synthesized via a solid-phase reaction typically contains large agglomerates and exhibits low sinterability. The modified B-site precursor method adopted in this study overcame these shortcomings and enabled the preparation of monophasic ZT solid solutions with various Z/T ratios [20,21].

#### 3.1.2. Metastable Perovskite-Type PZT Solid Solutions

Perovskite-type PZTN solid solutions with the formula Pb_0.99_(Zr_0.54+*x*_Ti_0.46_)_0.98_Nb_0.02_O_3+1.96*x*_ (*x* = 0–0.04) were synthesized using ZT as the B-site precursor in a solid-phase reaction with PbCO_3_. A significantly lower temperature of 740 °C was employed using this approach.

**Figure 3:**
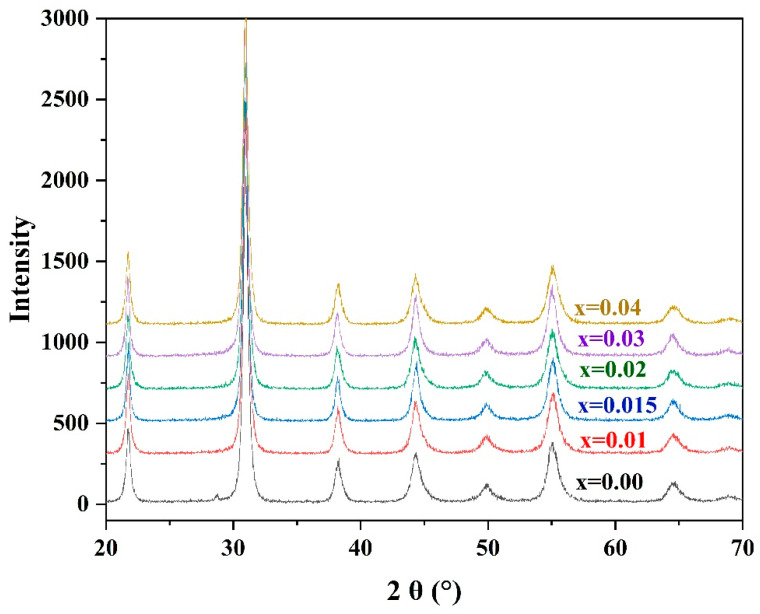
XRD patterns of PZTN powders synthesized at 740 °C for 4 h.

The XRD patterns of the ceramic powders synthesized from the PZTN powders at 740 °C for 4 h (Figure 3) reveal the existence of a ferroelectric rhombohedral phase. The patterns were consistent with the standard JCPDS XRD data for PZT with the formula Pb(Zr_0.58_Ti_0.42_)O_3_ (JCPDS card No. 73–2022), indicating that the PZT ceramic powders synthesized at 740 °C were monophasic without any PbO impurities, PbTiO_3_, or PbZrO_3_ intermediate phases.

As the radius of Zr^4+^ (7.9 nm) is greater than that of Ti^4+^ (6.8 nm), an increase in the Zr^4+^ content of the unit cell should increase the size of the unit cell. Bragg’s law (2*d*sinθ = λ) indicates that the diffraction peak should be shifted towards smaller angles; however, no such shift was observed in the peak characteristic of PZT with an increasing ZrO_2_ content (Figure 2). This indicates that the size of the prepared metastable perovskite-type unit cells did not increase with the addition of ZrO_2_. Such a phenomenon can be explained by the fact that the number of Pb vacancies increases with increasing ZrO_2_ content, which usually reduces the size of the unit cell.

Using the procedure described in Section 2.1, single-phase perovskite-structured PZT solid solutions with an A-site: B-site stoichiometric ratio of 1:1 was synthesized at temperatures as low as 725 °C. Although the synthesis of perovskite solid solutions with Pb vacancies required a temperature of 740 °C, this is significantly lower than that required using traditional solid-phase reactions (850 °C) [23] or the organic polymer synthesis method (800 °C) [24] used in the synthesis of monophasic PZT powders. This reduction in temperature significantly reduces the extent of PbO volatilization during the procedure.

#### 3.1.3. Intragranular PZT/ZrO_2_ Nanocomposite Ceramics

A non-stoichiometric A-site: B-site ratio of less than 1 was observed in the perovskite lattice of the Pb_0.99_(Zr_a+*x*_Ti_1-a_)_0.98_Nb_0.02_O_3_ solid solution. The relevant phase diagram [25] shows that the solid solution of such a composition is thermodynamically unstable and thus a more stable phase (PZT + ZrO_2_) is formed through the following reaction at higher sintering temperatures:

PbZr_1−*x*+a_Ti*_x_*O_3_ → PbZr_1−*x*_Ti*_x_*O_3_ + aZrO_2_ (5)

The ceramic sintering temperature varies according to the particle size, composition, and the state of the formed powder. The temperature of martensitic transformation (*t*-ZrO_2_→*m*-ZrO_2_) is 1170 °C, so the sintering temperature must be higher than 1170 °C, the sintering compactness of PZT ceramics should also be considered. In this study, the sintered temperature of 1260 °C was determined by sintering tests with the density measurement of the specimen. A high temperature ramp rate from 900–1000 °C enabled faster grain growth and grain boundary movement, which favored the encapsulation of smaller modified oxide grains by PZT grains to form intragranular grains. As these grains significantly influence the mechanical properties and internal stress of the material, the temperature ramp rate will therefore affect the mechanical properties of the samples to a certain extent. A ramp rate of 7 °C min^−1^ from 900–1000 °C was adopted during the sintering process to form more intragranular structures.

### 3.2. SEM Analysis of PZT/ZrO_2_ Nanocomposite Ceramics

#### 3.2.1. SEM Analysis of Natural Surfaces

**Figure 4:**
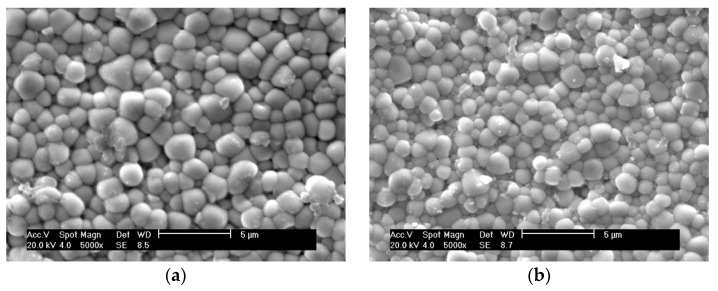

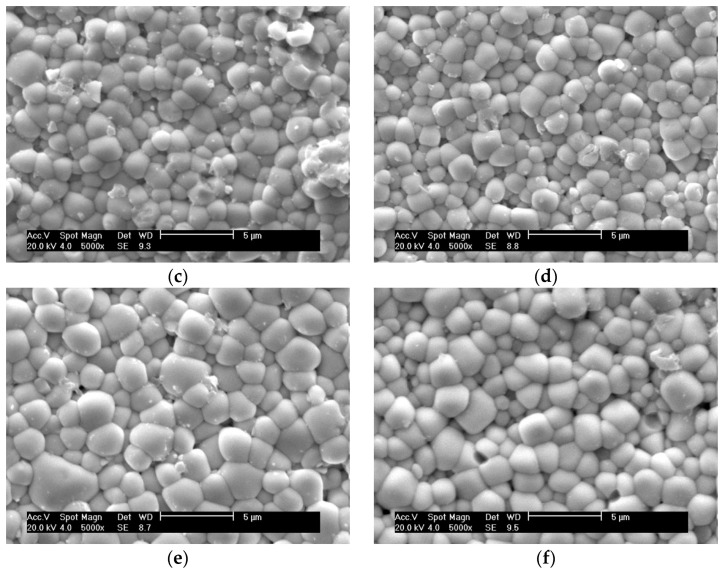
SEM images of the natural surfaces of PZTN nanocomposite ceramics with (**a**) *x* = 0, (**b**) *x* = 0.01, (**c**) *x* = 0.015, (**d**) *x* = 0.02, (**e**) *x* = 0.03, (**f**) *x* = 0.04.

SEM images of the natural surfaces of PZTN composite ceramics sintered at 1260 °C for 2 h show that the sample obtained without ZrO_2_ (*x* = 0) had a mean grain size of <2.5 μm and exhibited plump, spherical grains (Figure 4a). The grains obtained at *x* = 0.01 were significantly smaller and more tightly bound to one another than those obtained at *x* = 0 (Figure 4b), which is consistent with the results reported by Malic et al. [8] and can be explained by the fact that the addition of ZrO_2_ inhibited the matrix grain growth via the Zener pinning effect [26].

The grain size increased gradually when increasing amounts of ZrO_2_. These results are not consistent with those reported in the literature [8], nor with the theory that grain growth is inhibited by the pinning effect produced in the matrix by the precipitation of nanoparticles in the secondary phase. This discrepancy is attributed to the presence of Pb vacancies in the metastable perovskite-type PZT powders, which increased with increasing amounts of ZrO_2_. As the A-site vacancies in the lattice aid in dispersion and promote sintering, further addition of ZrO_2_ caused substantial grain growth. The pinning effect exerted by the precipitated nanoparticles favored a reduction in the glass phase and reduced the number of pores, thereby strengthening the grain boundaries. The absence of impurities and the glass phase among the grains led to stronger grain bonding after the addition of ZrO_2_ (Figure 4b–f), thereby toughening the matrix. We believe that the joint action of the two effects described above contributed positively to the sintering of PZT/ZrO_2_ nanocomposite ceramics.

The determination of fracture modes and locations (i.e., intragranular or intergranular) of dispersed secondary-phase particles in the ceramic samples required further analysis of fracture morphologies and energy spectra.

#### 3.2.2. SEM Analysis of Fractured Surfaces

**Figure 5:**
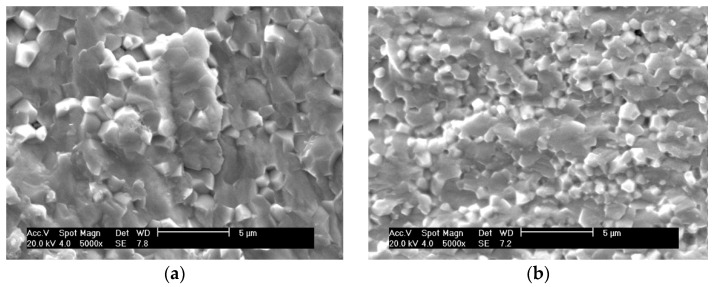

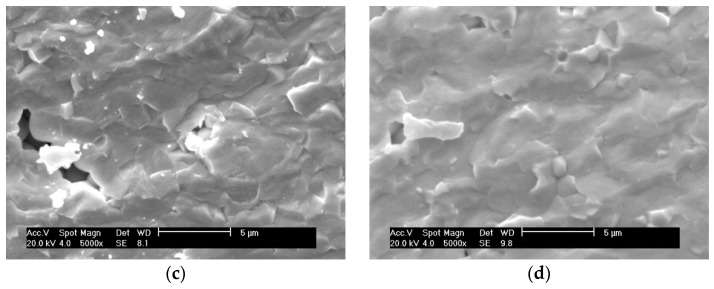
SEM images of the fractured surfaces of PZTN composite ceramics sintered at 1260 °C for 2 h with (**a**) *x* = 0, (**b**) *x* = 0.01, (**c**) *x* = 0.03, and (**d**) *x* = 0.04.

SEM images of the fractured surfaces of PZTN nanocomposite ceramics with various ZrO_2_ contents sintered at 1260 °C for 2 h show that an increase in the ZrO_2_ content changed the fracture mode from transgranular fracture to intergranular fracture (Figure 5), with the fracture mode being essentially transgranular when *x* = 0.04 (Figure 5d). This indicates that nanocomposite ceramics containing ZrO_2_ exhibit reinforced grain boundaries compared to pure PZT ceramics, significantly increasing both the fracture strength and toughness. The SEM images revealed that large amounts of intergranular impurities and glass phase content were absent, which is in agreement with the SEM images of the natural surfaces. ZrO_2_ particles were almost completely absent between the grain boundaries of the PZT matrix, which differs from the reported observations of previous studies that found dispersed ZrO_2_ particles between grain boundaries [27,28,29,30]. These results indicate that the prepared materials were intragranular nanocomposite ceramics.

As the increasing of ZrO_2_ addition, the amount of ZrO_2_ particles dispersed in PZT matrix increased.

Due to the stress field generated by the second phase ZrO_2_ particles in the PZT ceramic matrix, the PZT matrix will be toughened by residual stress. The residual stress introduced by the ZrO_2_ became stronger and stronger, this residual stress can effectively absorb the energy and increase the crack extends resistance [28,29,30], which reduced the sensitivity toward crack and weakened the tense concentration effect around the point of the crack, resulting in the fracture morphology change of transgranular fracture to intergranular fracture, and the intergranular fracture meaning the improvement of the cracking resistance

### 3.3. TEM and EDS Analyses

#### 3.3.1. Nanosized ZrO_2_ Particles

**Figure 6:**
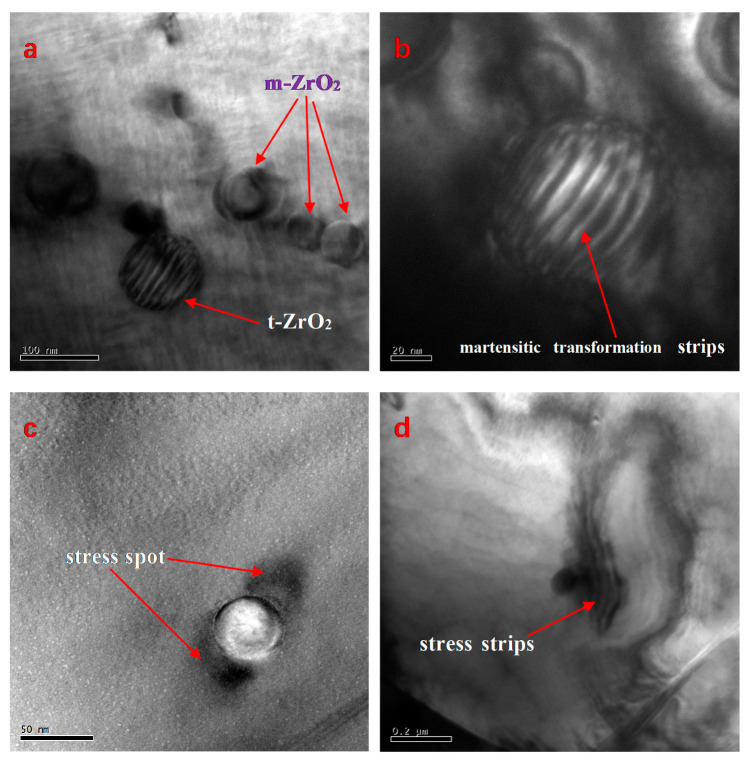
TEM images of the PZTN (*x* = 0.04) composite ceramic sintered at 1260 °C for 2 h. (**a**) Intragranular monoclinic and tetragonal ZrO_2_ spheres; (**b**) Dark-field image of martensitic transformation (striped pattern); (**c**) Stress spots around a ZrO_2_ sphere; (**d**) Stress stripes around a ZrO_2_ nanoparticle.

TEM images of the PZTN (*x* = 0.04) nanocomposite ceramic sintered at 1260 °C for 2 h and subsequently subjected to particle thinning reveal the presence of monoclinic (*m*-ZrO_2_) and tetragonal (*t*-ZrO_2_) intragranular ZrO_2_ nanospheres (Figure 6), with m-ZrO_2_ being smaller (diameter < 50 nm) than *t*-ZrO_2_ (~100 nm). A martensitic transformation can be observed on one *t*-ZrO_2_ sphere, whereas a smaller t-ZrO_2_ sphere next to the larger one did not undergo a phase transition during the cooling process (Figure 6a). Zr^4+^ ions tend to form monoclinic structures (coordination number < 8) at low temperatures, whereas cubic and tetragonal forms with Zr-O_8_ coordination structures can are only stable at high temperatures owing to vibrational equilibrium. The *t*-ZrO_2_→*m*-ZrO_2_ transition is a martensitic transformation characterized by a first-order phase transition, thermal hysteresis (i.e., the phase transition occurs within a certain temperature range),) a change in volume of approximately 3–5%, and a change in shear strain of 1–7% during phase transition. The tetragonal phase is maintained by the smaller particle size and the compressive stress exerted by the PZT matrix. This is a necessary condition for the phase transition of ZrO_2_ and matrix toughening in PZT/ZrO_2_ nanocomposite ceramics. Stress spots formed by the exertion of forces by monoclinic ZrO_2_ on the matrix and martensitic transformation stripes on *t*-ZrO_2_ are clearly visible in the dark-field image of ZrO_2_ nanoparticles (Figure 6c). The presence of stress spots around a single monoclinic ZrO_2_ sphere (Figure 6c) and ZrO_2_ spheres (diameter ~100 nm) with clear outward-propagating stress stripes (Figure 6d) will affect the macroscopic fracture mode of ceramic materials.

Residual stress in PZT/ZrO_2_ composite ceramics is generated via the following mechanism: a martensitic transformation occurs in the *t*-ZrO_2_ particles, causing a thermal mismatch between the ZrO_2_ particles and PZT, leading to residual stress within the PZT matrix and ZrO_2_ particles. A comparison of the TEM images in Figure 6c,d reveals that volume expansion caused by the martensitic transformation in *t*-ZrO_2_ during the cooling process resulted in significantly greater stress between the two phases of the material. Energy is effectively absorbed by the residual stress, thereby inhibiting crack propagation. The reduction in sensitivity toward cracks and weakening of the stress concentration effect at crack tips are key mechanisms, by which ceramic materials are toughened.

#### 3.3.2. Ferroelectric Domains in PZT/ZrO_2_ Nanocomposite Ceramics

**Figure 7:**
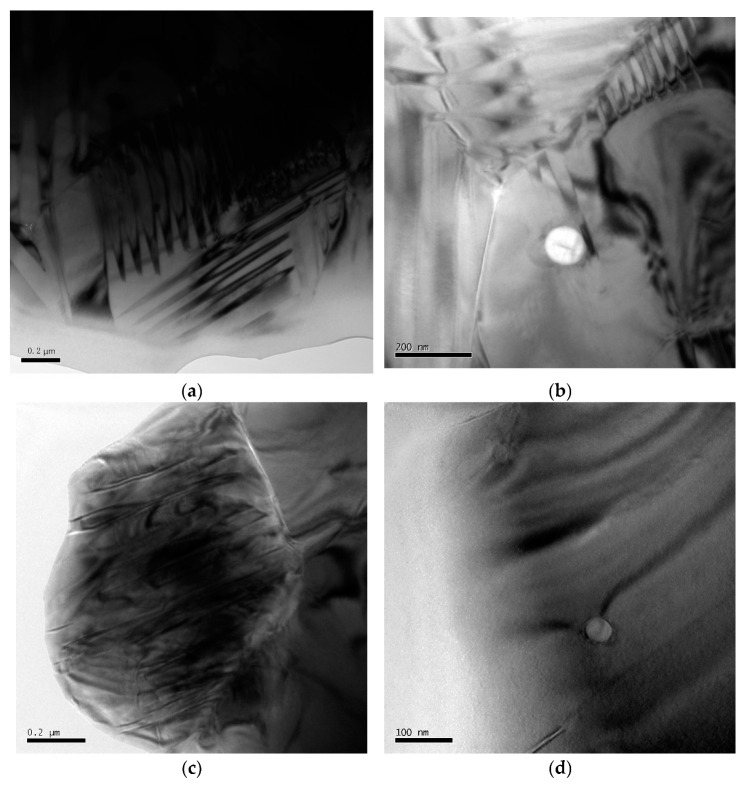
TEM images of ferroelectric domains in PZT/ZrO_2_ (PZTN, *x* = 0.04) nanocomposite ceramics sintered at 1260 °C for 2 h. (**a**) 90° domain; (**b**) Effects of ZrO_2_ on 90° domain; (**c**) 180° domain; (**d**) Effects of ZrO_2_ on 180° domain.

In ferroelectric materials, the spontaneous polarization in each domain is oriented in the same direction. The type of ferroelectric domain structure is primarily determined by the symmetry of the crystal structure before the ferroelectric phase transition and the orientation of spontaneous polarization [31]. The most common domain configurations in ferroelectric ceramics are the 90° and 180° domains. TEM images of the ferroelectric domains in the PZT/ZrO_2_ nanocomposite ceramics sintered at 1260 °C for 2 h show that an in-plane 90° domain is formed when adjacent polarization directions are perpendicular to each other (Figure 7a,b); when they are antiparallel to each other, an out-of-plane 180° domain is formed (Figure 7c,d). 90° and 180° domains are known to be situated on different crystal surfaces: 90° domains appear as a linear striped contrast pattern often with herringbone or dagger-like morphology [32], whereas 180° domains manifest as an irregular water mark-like or sinuous stripe contrast pattern [31].

The piezoelectric properties of PZT ceramics are determined by ferroelectric domain inversion during the polarization process. As PZT piezoelectric ceramics have a lower *K*_IC_, cyclic electric field loading on the material during its use can lead to microcrack formation and stable crack propagation visible to the naked eye. This can ultimately lead to the fracture, deterioration, and destruction of the material properties, owing mainly to structural changes in the ferroelectric ceramics induced by repeated domain inversion (i.e., domain switching) under the effects of an externally applied alternating electric field [32,33]. Therefore, methods that inhibit crack propagation and enhance toughness are imperative to preserve the properties and prolong the service life of PZT ceramics. Secondary-phase ZrO_2_ nanoparticles can effectively inhibit crack propagation (Section 3.3.2), while the phase transition of metastable tetragonal ZrO_2_ also provides toughening effects. As the size of ferroelectric domains is of the order of nanometers or submicron-sized domains, ZrO_2_ nanoparticles dispersed within the PZT matrix will undoubtedly exert a significant influence on the domains in the material. The effects of ZrO_2_ nanospheres on the domains include domain truncation (Figure 7b) and deflection (Figure 7b,c), which absorb and reduce the energy that causes domain inversion. Therefore, dispersed ZrO_2_ nanoparticles can concurrently inhibit crack propagation and directly prevent potential domain inversions caused by crack formation [34].

**Figure 8:**
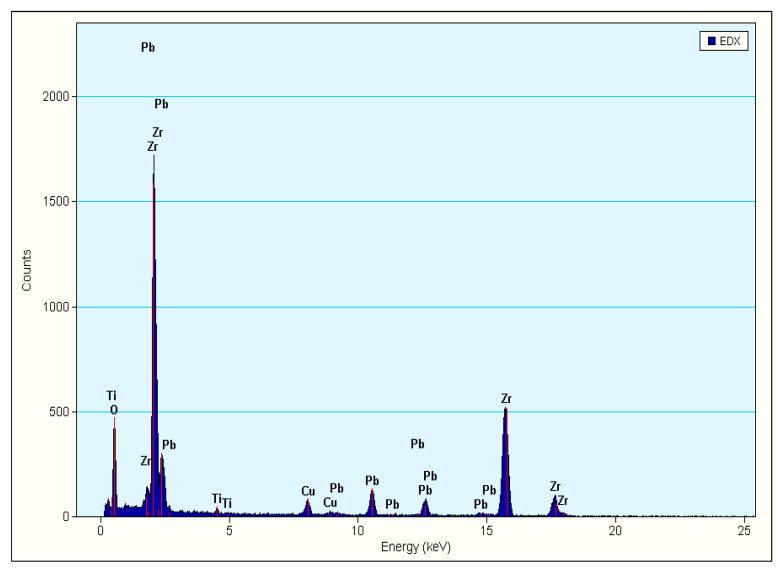
EDS of TEM of the PZTN (*x* = 0.04) sintered at 1260 °C for 2 h.

Figure 8 shows the EDS analysis of nano-sized ZrO_2_ spheres during the above TEM analysis. As shown in Figure 8, the peak of the Zr element is the strongest. The qualitative results are shown in Table 1. The atomic content of zirconium accounts for 88.81 wt% of the total metal atoms, which shows that these spheres (shown in Figure 6) are indeed ZrO_2_ particles. Using field emission transmission electron microscope to observe the existing position and the state of ZrO_2_ is a common method in the study of nano multiphase ceramics with ZrO_2_ as toughening phase [28,35,36,37].

### 3.4. Measured and Relative Densities

The theoretical densities of the PZT matrix and ZrO_2_ are 8.00 kg m^−3^ and 5.83 kg m^−3^, respectively [8]. The measured density of the PZT matrix (7.78 kg m^−3^ at *x* = 0) decreased with increasing ZrO_2_ content, reaching a value of 7.53 kg m^−3^ at *x* = 0.04 owing mainly to the lower density of the secondary-phase ZrO_2_ compared to the matrix. Precipitated ZrO_2_ with a higher m-ZrO_2_ content would cause a greater reduction in the measured density. All sintered samples had relative densities of at least 95%, demonstrating that sintering at 1260 °C for 2 h afforded optimum mechanical, dielectric, and piezoelectric properties [36]. 

The electrical properties of ceramics are closely related to the compactness of ceramics, high density in sintered ceramics effectively reduces internal frictional losses and enhances volume resistivity, which greatly improves their dielectric and piezoelectric properties.

### 3.5. Dielectric Properties

#### 3.5.1. Dielectric Constant and Dielectric Loss

**Figure 9:**
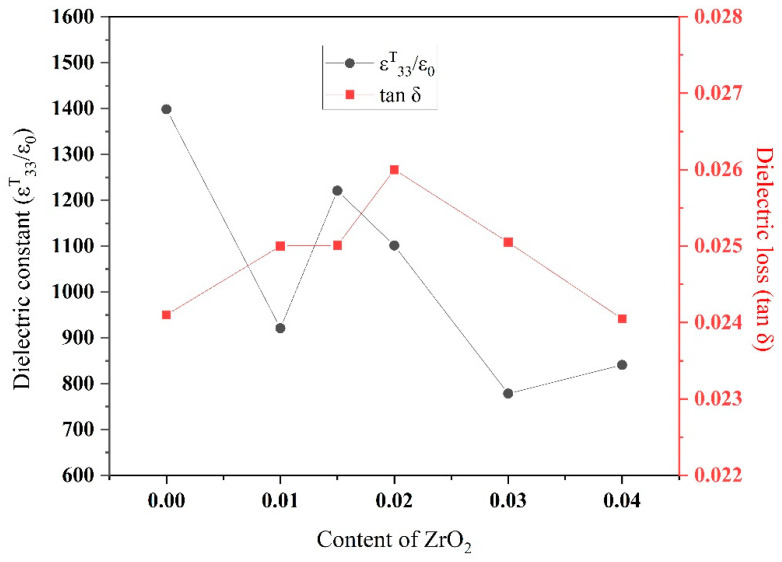
Variation in ε*^T^*_33_/ε_0_ and tan *δ* with ZrO_2_ content in PZTN nanocomposite ceramics sintered at 1260 °C for 2 h.

Figure 9 shows the variation in dielectric constant and tan *δ* as a function of ZrO_2_ content in PZTN composite ceramics sintered at 1260 °C for 2 h. Tan *δ* initially increased as the ZrO_2_ content increased to 0.02, and subsequently decreased with increasing ZrO_2_ content. The maximum tan *δ* of approximately 0.026 was recorded at *x* = 0.02. The dielectric constant exhibited an overall downward trend with increasing ZrO_2_ content. Both the dielectric constant and tan *δ* are related to ceramic density, with denser ceramics exhibiting higher dielectric constants. Tan *δ* is widely thought to be related to domain wall movement. Inter-domain wall, domain wall–lattice, and domain wall–defect interactions occur upon application of an external electric field, causing energy losses during domain wall movement. A lower density typically results in increased losses during domain wall movement. Grain growth increases the sizes of the 180° and 90° domains, generating appreciable stress owing to a change in the orientation of the 90° domain, thereby increasing dielectric loss. In addition, an increase in the content of secondary-phase ZrO_2_ increases the generated internal stress and direct effects of ZrO_2_ particles on the domains (Figure 7), which also contributes to high stresses during changes in domain orientation.

#### 3.5.2. Temperature Stability and Curie Temperature

**Figure 10:**
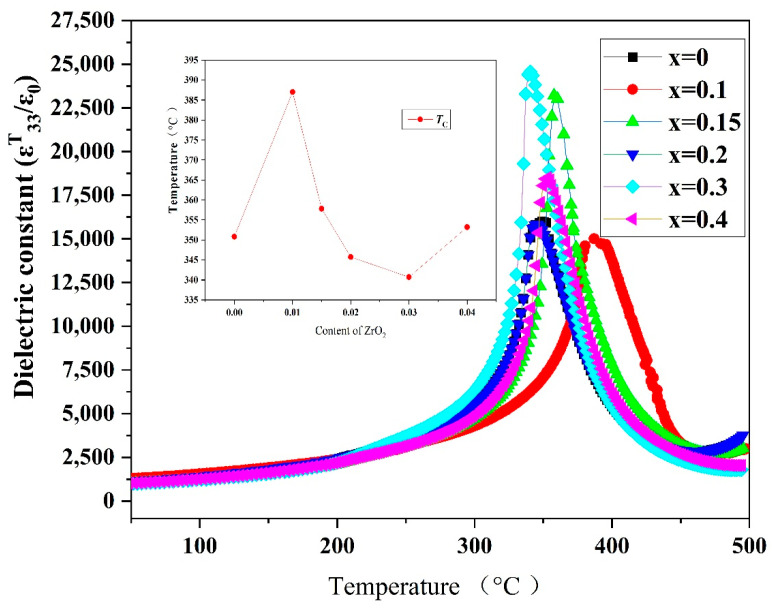
Dielectric thermograms (1 kHz) of PZTN ceramics sintered at 1260 °C for 2 h.

Figure 10 shows the variation in *ε^T^*_33_/*ε*_0_ (at 1 kHz) with in PZTN ceramics with various ZrO_2_ contents as a function of temperature. The Curie temperature (*T_c_*) of the ceramics initially increased and subsequently decreased with increasing ZrO_2_ content. At *x* = 0.01, the highest *T_c_* value of 387 °C, along with the lowest *ε^T^*_33_/*ε*_0_ was obtained at *x* = 0.01. At *x* = 0.03, the lowest *T_c_* value of 340.7 °C was obtained; however, the highest *ε^T^*_33_/*ε*_0_ value was achieved. The peak *ε^T^*_33_/*ε*_0_ values of the PZTN ceramics were in the range of 12,000–25,000.

The variation in the *ε^T^*_33_/*ε*_0_ of ferroelectric materials with temperature is mainly caused by the influence of temperature on spontaneous polarization. Piezoelectric ceramics exhibit ferroelectricity and paraelectricity below and above *T_c_*, respectively. The dielectric constant of a material typically peaks at the ferroelectric-paraelectric phase transition point (Curie point). At temperatures below *T_c_*, the dielectric constant decreases with temperature in the absence of any phase transition. At temperatures below the Curie point, the dielectric constant increased with increasing temperature (Figure 10). 

Ferroelectricity in piezoelectric ceramics is characterized by an increase in the dielectric constant with temperature. When the temperature surpasses a certain value, a ferroelectric to paraelectric phase transition occurs, causing a sharp reduction in the dielectric constant. The corresponding phase transition temperature is known as the Curie temperature (*T*_c_), whose magnitude is critical in high-temperature piezoelectric materials.

**Figure 11:**
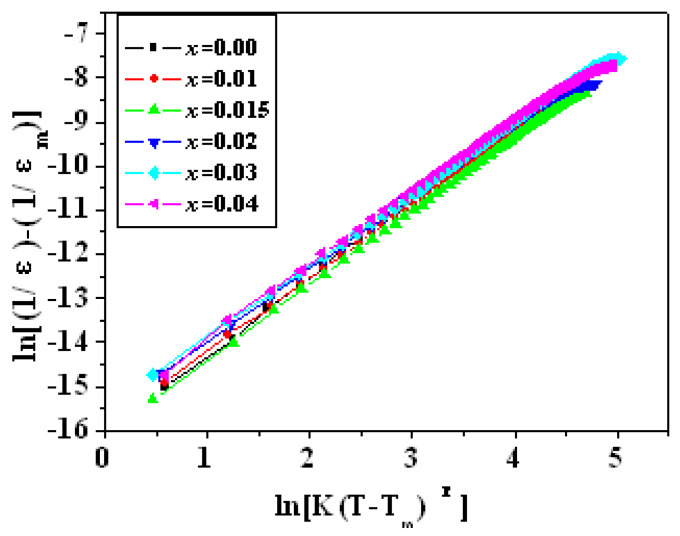
Variation in the reciprocal of *ε* with *T − T*_m_ in PZTN ceramics sintered at 1260 °C for 2 h.

The variation in the reciprocal of *ε* with T − T_m_ in all samples of PZTN ceramics sintered at 1260 °C for 2 h, followed the relationship: (1/*ε*) − (1/*ε*_m_) = K(T − T_m_)^γ^, with *ε* values being higher than those at *T*_c_ (Figure 11). All γ values (Table 2) were higher than that in the Curie-Weiss law for normal ferroelectrics (γ = 1.0) and smaller than that in the Curie-Weiss law for relaxor ferroelectrics, such as PMN (γ = 2.0) [37]. The γ values initially decreased, then increased and decreased again with increasing ZrO_2_ content.

#### 3.5.3. Dielectric Loss at Different Temperature

**Figure 12:**
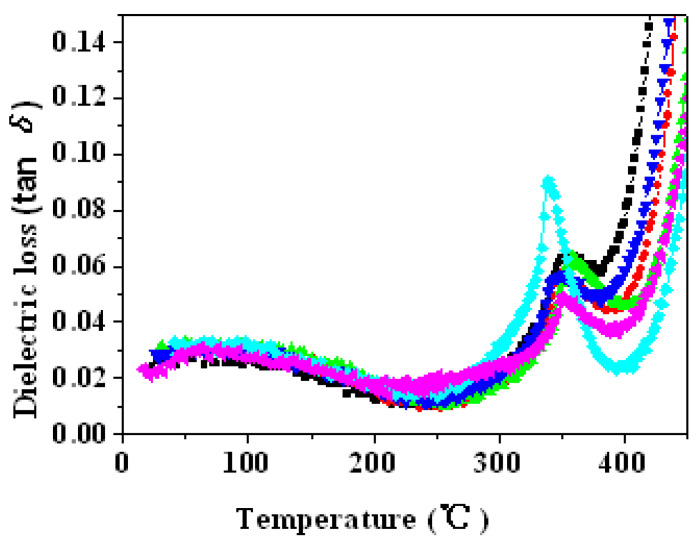
Variations in dielectric loss (1 kHz) with temperature in PZTN ceramics sintered at 1260 °C for 2 h.

A plot of the variations in tan *δ* (at 1 kHz) in PZTN ceramic samples sintered at 1260 °C for 2 h as a function of temperature (Figure 12) reveals that tan *δ* initially decreased with increasing temperature owing to the absorption of moisture by the samples exposed to an open-air environment. The maximum value of tan *δ* was achieved near *T*_c_, similar to *ε*.

Dielectric loss arises from conductivity and relaxation polarization. In piezoelectric ferroelectrics, conductivity loss is negligible and dielectric loss is mainly caused by relaxation polarization. At low temperatures, the dipole moment remains essentially unchanged with changes to an external electric field, leading to a small relaxation loss. As the temperature increases, increased thermal motion of the dipole moment induces higher dielectric loss. In ferroelectric ceramics, dielectric loss is also related to domain wall movement. Elevated temperatures increase domain wall movement, thereby increasing dielectric loss [36].

### 3.6. Piezoelectric Properties

**Figure 13:**
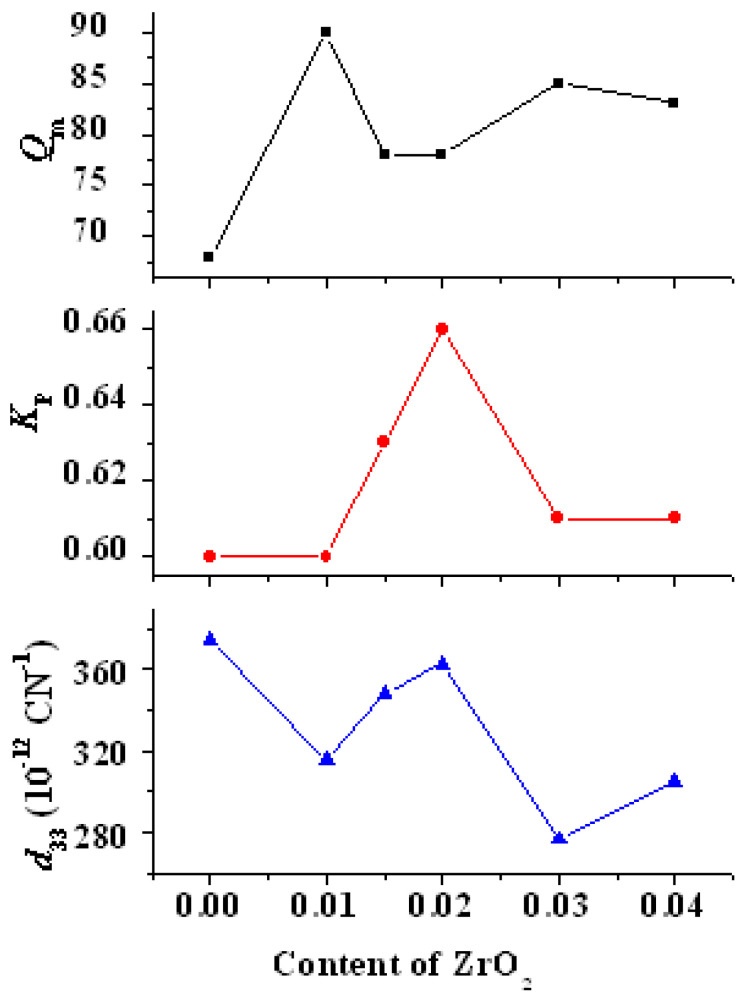
Variations in *d*_33_, *K*_p_, and *Q*_m_ with ZrO_2_ content in PZTN ceramics.

Figure 13 shows the variations in *d*_33_, *K*_p_, and *Q*_m_ with ZrO_2_ content in PZTN ceramics sintered at 1260 °C for 2 h. The *K*_p_ and *Q*_m_ of all samples containing ZrO_2_ were higher than those of the samples without ZrO_2_. *K*_p_ reached a maximum value of 0.66 at *x* = 0.02, whereas *Q*_m_ reached a maximum of 90 value at *x* = 0.01. *d*_33_ exhibited an overall downward trend, with a relatively high value of 363 pC N^−1^ at *x* = 0.02. *Q*_m_ reached a minimum value of approximately 80 when *d*_33_ and *K*_p_ reached their maximum values. *Q*_m_ decreased with increasing porosity because it is determined by the relaxation loss of the domain walls, which is primarily caused by domain wall movement (especially during polarization). The presence of an internal bias field is not conducive to changes in the domain wall orientation and makes polarization difficult, thereby favoring high *Q*_m_ values. Sn increase in *K*_p_ and *d_3_*_3_ typically leads to a reduction in *Q*_m_.

The results depicted in Figure 13 show that the piezoelectric properties of PZTN ceramics did not strictly decrease with increasing ZrO_2_ content as reported in the literature [23], with *K*_p_, *Q*_m_, and *K*_31_ showing both increasing to various extents. In addition, *d*_33_ at *x* = 0.02 is very similar to that at *x* = 0.

## 4. Conclusions

A ZT oxide solid solution of B-site ions was synthesized within perovskite-type PZT via a precursor method. The temperature required by this procedure was significantly reduced to 740 °C from the 1000 °C required by traditional solid-phase reactions. The synthesized solid solution was subsequently employed as the B-site precursor in the synthesis of metastable PZT powders with Pb vacancies at the A-sites via a solid-phase reaction with PbCO_3_. XRD analysis of the PZT powders revealed monophasic perovskite structures, which yielded PZT/ZrO_2_ piezoelectric ceramics upon sintering. The intragranular niobium-doped PZT/ZrO_2_ nanocomposite structure of the fabricated PZT/ZrO_2_ ceramics was confirmed by XRD, SEM, and TEM analyses. 

The fracture mode of the nanocomposite ceramics changed from intergranular to transgranular fracture with increasing ZrO_2_ content, while grain size showed an initial reduction followed by an increase. The temperature ramp rate during sintering was increased to enable the precipitation of PZT unit cells for the preparation of PZT/ZrO_2_ nanocomposite ceramics. TEM analysis revealed the presence of both tetragonal and monoclinic ZrO_2_ phases in the nanocomposite ceramics. TEM images also showed that secondary-phase ZrO_2_ nanoparticles primarily existed within the PZT grains and grain boundaries, while tetragonal ZrO_2_ particles measuring < 50 nm were present within the PZT/ZrO_2_ nanocomposite ceramics. Additionally, ZrO_2_ exerted truncating and deflecting effects on ferroelectric domains. 

The piezoelectric properties of the nanocomposites increased to various extents with increasing ZrO_2_ content. The optimal piezoelectric properties of PZTN nanocomposite ceramics (*ε^T^*_33_/*ε*_0_ = 1398, tan *δ* = 0.024 *d*_33_ = 354 pC N^−^^1^, *k*_p_ = 0.66 *Q*_m_ = 78) are obtained when *x* = 0.02. *T_c_* increased and subsequently decreased with increasing ZrO_2_ content, with the highest value of *T*_c_ (387 °C) and the lowest value of *ε^T^*_33_/*ε*_0_ (15010) being obtained at *x* = 0.01.

## Figures and Tables

**Table 1:** Elements analysis by EDS of the PZTN (*x* = 0.04) sintered at 1260 °C for 2 h.

Element	Weight %	Atomic %	Uncertainty %	Correction	k-Factor	Correction
O(K)	8.922	38.361	0.199	0.495	2.059	0.867
Ti(K)	0.689	0.989	0.023	0.993	1.290	0.993
Zr(K)	72.595	54.742	0.582	0.999	3.930	0.999
Pb(L)	17.792	5.906	0.342	0.753	6.528	0.999

Correction method: Thickness Detector Absorption.

**Table 2:** Diffusion coefficients (γ) of PZTN ceramics sintered at 1260 °C for 2 h.

Secondary-Phase ZrO_2_ Content	γ (1 kHz)
*x* = 0.00	1.70
*x* = 0.01	1.67
*x* = 0.015	1.66
*x* = 0.02	1.58
*x* = 0.03	1.61
*x* = 0.04	1.58

## Data Availability

The data used to support the findings of this study are included within the manuscript.
